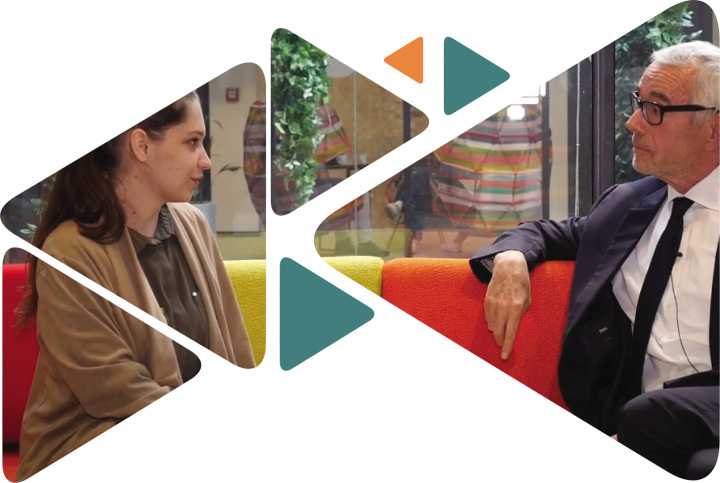# Johannes Vester – Adapted interviews from Neurotrauma Treatment Simulation Center (NTSC) – Vienna, 2022

**DOI:** 10.25122/jml-2022-1018

**Published:** 2022-07

**Authors:** Alexandra-Mihaela Gherman, Stefana-Andrada Dobran, Andreea Strilciuc

**Affiliations:** 1. RoNeuro Institute for Neurological Research and Diagnostic, Cluj-Napoca, Romania; 2. Sociology Department, Babes-Bolyai University, Cluj-Napoca, Romania

**Interviewee: Johannes VESTER**

**Interviewer: Stefana DOBRAN**

**S.D**.: Can you tell us a few words about yourself?

**J.V**.: As the president of the AMN (the Academy for Multidisciplinary Neurotraumtology), it's my great pleasure to offer a kind of umbrella for this NTSC (Neurotrauma Treatment Simulation Center) meeting because it bridges high science with what happens really down to earth, namely what happens in the treatment of the patient. We have to establish a kind of feedback cycle from research, scientific mindset and to the practice, but also the feedback from there. So, this event is bridging this and bringing life into the cycle so that there are no isolated scientific researchers and isolated people just working locally, in the local conditions, with the local obstacles, with the patient, but to get their living interchanged and communication between this. And this is what this whole program, which is a fantastic program, is trying to establish here for the first time.

**S.D**.: Right, so it's an integrated bottom-up and top-down approach.

**J.V**.: You said it. Exactly, yes!

**S.D**.: Could you describe how your experience shaped your opinion regarding the treatment of neurotrauma?

**J.V**.: I was involved in the first large-scale TBI trial in Germany, [a] multidisciplinary large-scale trial. The group was responsible at that time for the study design and, unfortunately, I saw a lot of trials failed. Most trials actually failed and there were reasons for that. But, as in many other domains, you just repeat the way people are doing trials instead of looking at why they fail. A failed study is where we have to get to work. We have to find out why [...] And today we have so much more knowledge behind the curtain of how to produce effectively clinical research, and how to control the chances.

By doing this we get more insights, we can detect new treatments and it's important to know about the framework. And this is also one part of this NTSC meeting, to enlighten the view behind where research is coming from and how we can contribute to these conditions.

**Figure:**
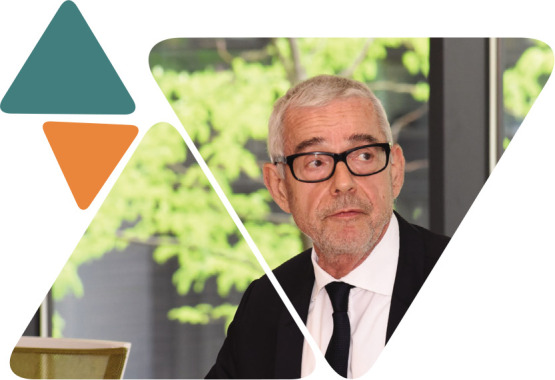


**S.D**.: If you had to identify challenges in the approach of TBI research in current times, which three (3) of them would come to mind?

**J.V**.: I would say, first of all, it's not learning from the past.So, we have to learn from the failures, we have to lose the anxiety.[...] One of the problems is the heterogeneity of the population.[...] In TBI, in most cases you will have no baseline. So, you only have the values after one (1) month, after nine (9) months, after one (1) year and all the heterogeneity of the population of the degree of severity goes fully into the outcomes scale. So, it's really a problem how to get the comparability of the groups and there are some fantastic possibilities nowadays to deal with this, so this is one of the problems. The other problem is the still too large separation between the departments. At the end there is a patient, a human being[...] and this whole voyage includes neurosurgeons, neurologists, and neurorehabilitation, so we have to develop a new mindset that we are accompanying the patient across this whole process. [...] We have to put away the academic borders, that we enrich our knowledge and that we share this knowledge and then we get completely new aspects of how to treat patients maybe very early to prevent something like depression [...]. So, this integration of the pathway is one of the very important things for the future, and, also, one of the tasks here (at NTSC) [...] bringing all these domains and disciplines together, also from the AMN, as multidisciplinary approach. It's so important!

**S.D**.: Thank you! From your point of view, what would be the main limitations in the approach of cognitive, behavioral or depressive disorders resulting from neurotrauma? Out of your answer before.

**J.V**.: Basically, it's already what I said before. [...] So, it's important that we all learn from each other, that all disciplines learn from each other, and it's rather new that there are so many cognitive problems and also mood problems, emotional problems which may-like depression-develop [...] So, this awareness of these later problems is just [like] emerging in the mindset of all clinicians and researchers in neurotrauma. One did not know that there are so many problems, cognitive problems, coming in the later stage and so it's important to see this is a consequence [...] of the original trauma and we have from the very beginning to keep this in mind and to think about what we can do to prevent that.

**S.D**.: Alright. I have actually an additional question on this topic. So, how do you see the impact of a TBI registry over the management of neurotrauma?

**J.V**.: Yes, it's one of the answers of what I just said before: to get knowledge about this. [...] So, there is sometimes a huge gap to get the information-how many [patients], how do the patients develop and one of the big values is [...] to observe patients in development across time. [...] We get another feeling of what happens throughout this whole development after the trauma. So, the registry is [about] giving the facts, the data [...]. So it makes sense to prevent something in advance, which is always cheaper than later on repairing things.

**S.D**.: Online communities in the medical field are becoming more and more visible.

Considering this, what do you think their role would be in medical practice today and research?

**J.V**.: I think it's of great value. It's different from in-person communication, other things happen. Online communication is for quick exchange of knowledge, quick exchange for an existing knowledge to generate new knowledge. Personal contact is very important, because you share directly your view with another person, with other scientists and then you develop something. It is difficult to do this online. [...] So, it has a great value for the future but it cannot compensate for the in-person coordinations and in-person meetings.

**S.D**.: And my last question would be, from your perspective, which are the obstacles that affect a proper long-term follow-up in cases of neurotrauma?

**J.V**.: [...] Let's say it's also part of the mindset. [...] Because there is still a gap of knowledge about the problems coming later on. [...] So, it makes sense to learn from the follow-up and to integrate the long-term follow-up into our whole mindset. [...]

So, it's very important in our definition

'What is a human being?'

'A human being is simply the same as before; a living person, but with social, emotional reintegration achieved [...]'

**S.D**.: Right. So, thank you very much Professor Vester for all your answers, [...] thank you for this program, and I hope to see many more in the future.

**J.V**.: Yes, it would be my pleasure. Thank you very much!

Watch the extended interview on the AMN Website: https://brain-amn.org/ntsc-interviews-series-johannes-vester-amn-president/

**Figure:**